# Matrine inhibits cell growth, migration, invasion and promotes autophagy in hepatocellular carcinoma by regulation of circ_0027345/miR-345-5p/HOXD3 axis

**DOI:** 10.1186/s12935-020-01293-w

**Published:** 2020-06-16

**Authors:** Shaobing Lin, Jie Zhuang, Liping Zhu, Zongsheng Jiang

**Affiliations:** 1 Department of Pharmacy, Fujian Provincial Hospital, Fuzhou, China; 2 Edinburgh University Joint Institute of Zhejiang University, Zhejiang University, Hangzhou, Zhejiang China

**Keywords:** Hepatocellular carcinoma, Matrine, circ_0027345, miR-345-5p, HOXD3

## Abstract

**Background:**

Matrine has been reported to exert anti-tumor effects in multiple types of cancers containing hepatocellular carcinoma (HCC). However, the anti-tumor molecular mechanisms of matrine in HCC is still not fully revealed.

**Methods:**

Cell viability, apoptosis, cycle, migration and invasion were determined by Cell counting kit-8 (CCK-8), Flow cytometry and Transwell assays, respectively. Levels of all protein were analyzed by western blot analysis. The levels of circular RNA_0027345 (circ_0027345), microRNA-345-5p (miR-345-5p) and homeobox-containingD3 (HOXD3) were detected by quantitative real-time polymerase chain reaction (qRT-PCR). The interaction between circ_0027345 and circ_0027345 was identified using dual-luciferase reporter assay. The mouse xenograft model was constructed to explore the effect of matrine on tumor growth in vivo.

**Results:**

Matrine suppressed cell growth, migration and invasion, while promoted apoptosis and autophagy in HCC cells. Matrine down-regulated the levels of circ_0027345 and HOXD3, and up-regulated miR-345-5p expression. Besides, circ_0027345 overexpression could reverse the inhibitory effect of matrine on cell progression. As the target gene of circ_0027345, miR-345-5p elevation counteracted the promotion effect of circ_0027345 overexpression on development of HCC cells. Moreover, miR-345-5p knockdown could facilitate cell growth, migration, invasion and repress cell apoptosis and autophagy by targeting HOXD3. Meanwhile, matrine restrained tumor growth of HCC by regulating circ_0027345/miR-345-5p/HOXD3 axis in vivo.

**Conclusion:**

Matrine inhibited cell development and tumorigenesis in HCC by increasing miR-345-5p and decreasing circ_0027345 and HOXD3.

## Highlights

- Circ_0027345 overexpression can reverse the effects of matrine on cell viability, migration, invasion and autophagy in hepatocellular carcinoma.
- Circ_0027345 can act as miR-345-5p sponge to regulate HOXD3 expression.
- Matrine inhibits the progression of hepatocellular carcinoma by regulating the circ_0027345/miR-345-5p/HOXD3 axis in vitro and in vivo.

## Background

Hepatocellular carcinoma (HCC) is a malignant tumor of the digestive system with a high mortality rate, accounts for 90% of primary liver cancers and is the third leading cause of cancer-related mortality globally [1, 2]. Transplantation is the most effective method for HCC treatment, however, due to the recurrence rate and high metastasis rate of the tumors during the transplantation process, advanced patients over 70% cannot receive transplantation [3]. Thus, exploiting novel and effective drugs for HCC treatment is urgent.

Matrine, an alkaloid extracted from the leguminous plant sophora flavescens, a traditional Chinese medicine, has been revealed to exhibit multiple pharmacological effects, including diuretic, antiviral, anti-allergic and anti-inflammatory effects [4, 5]. In addition, matrine has been found to have anti-tumor effect in a variety of cancers, such as melanoma [6], glioblastoma [7] and thyroid cancer [8]. The anti-cancer effect of matrine has also been reported in HCC, for example, matrine could suppress cell migration and invasion by modulating epithelial-mesenchymal transition in HCC [9]. However, there are few studies on how matrine plays an anti-tumor role in HCC, and the specific molecular mechanism is still unclear.

Circular RNAs (circRNAs) are highly stable non-coding RNAs due to their covalently closed loop structures [10]. In recent years, accumulating evidence has shown that circRNA plays an important role in tumor progression and gene regulation [11, 12]. In the study of Sun et al., they found that circ_0027345 was up-regulated in HCC tissues by circRNA microarray analysis, and this result was verified by qRT-PCR, which was consistent with the microarray results [13]. But, the function and molecular mechanism of circ_0027345 in HCC remain obscure. MicroRNA-345-5p (miR-345-5p) has been identified as an anti-cancer factor in human cancers, such as pancreatic cancer [14] and cholangiocarcinoma [15]. In HCC tissues and cells, miR-345 expression was down-regulated and its overexpression could inhibit cell metastasis [16]. Given the inverse expression pattern of circ_0027345 and miR-345-5p in HCC and the mechanism by which circRNA can act as a competing endogenous RNA (ceRNA) for miRNA to exert functions [17], we wondered whether there was a connection between circ_0027345 and miR-345-5p in HCC.

The genes of homeobox-containing (HOX) family are the major transcription factors for cell differentiation and morphogenesis during mammalian development, and they play a pivotal role in tumor genesis and metastasis [18, 19]. HOXD3 belongs to the third paralogous group of the HOXD gene family, it could regulate cellular motility and intercellular interactions to maintain cellular structural integrity [20]. Previous studies have shown that HOXD3 was aggrandized in multiple cancers and promoted cell proliferation and metastasis [21]. Importantly, HOXD3 could regulate the metastasis and angiogenesis of HCC cells [22]. While the involvement of HOXD3 in matrine-mediated anti-tumor processes in HCC has not been investigated. Here, we aimed to investigate the effects of matrine on cell growth, metastasis and autophagy in HCC, and figure out whether the mechanism of its action is related to circ_0027345, miR-345-5p, and HOXD3.

## Materials and methods

### Cell culture

Human HCC cell lines Huh-7 and HCCLM3 were purchased from Procell (Wuhan, China). The two cell lines were maintained in Dulbecco’s Modified Eagle Medium (DMEM, Invitrogen, Carlsbad, CA, USA) with 0.1% penicillin/streptomycin and 10% fetal bovine serum (FBS, Invitrogen) in a cell incubator at 37 °C with 5% CO_2_.

### Transfection

Overexpression plasmid of circ_0027345 (pcDNA-circ_0027345) and the control (pcDNA-NC), miR-345-5p mimic (miR-345-5p) and the control (miR-NC), inhibitor (anti-miR-345-5p) and the control (anti-miR-NC), small interference RNA targeting HOXD3 (si-HOXD3) and its control (si-NC) were acquired from GenePharma (Shanghai, China). These constructs were transfected into Huh-7 and HCCLM3 cells by using Lipofectamine 3000 (Invitrogen).

### Cell viability assay

96-well plates inoculated with Huh-7 and HCCLM3 cells were placed in a cell incubator overnight. Cells were then stimulated by different doses of matrine (0, 0.4, 0.8 or 1.6 mg/mL) for 48 h. Next, cells were washed and treated with 10 μL cell counting kit-8 (CCK-8, Beyotime, Shanghai, China) for another 2 h, and the optical density (OD) value was estimated by a Wellscan reader (Thermo Labsystems, Santa Rosa, CA, USA) at 450 nm.

### Cell apoptosis assay

Huh-7 and HCCLM3 cells were transfected or treated with matrine for 48 h. After cell collection, apoptotic cells were detected through an Annexin V fluorescein isothiocynate (FITC)/propidium iodide (PI) apoptosis detection kit (Beyotime). The cells were stained with 5 µL FITC and 5 µL PI for 15 min at 37 °C. Then cells were analyzed by flow cytometry (BD Biosciences, Franklin Lake, NJ, USA).

### Western blot

Proteins from HCC cells and nude mouse tumor tissues were extracted by RIPA solution (Beyotime) on the ice. Based on the molecular weight of the protein, different concentrations of sodium dodecyl sulfate–polyacrylamide gel electrophoresis (SDS-PAGE) was used to separate the proteins, and protein samples were then transferred to polyvinylidene difluoride (PVDF, Beyotime) membranes. Following blocking of 5% non-fat dry milk for 1 h, the membranes were treated with primary antibodies against B cell lymphoma-2 (Bcl-2, 1:1000, ab32124), Bcl-2-associated x (Bax, 1:2000, ab182733), Light chain 3-II/LC3-I (LC3-II/LC3-I, 1:1000, ab128025), Beclin 1 (1:1000, ab210498), HOXD3 (2 µg/mL, ab22840) or glyceraldehyde-3-phosphate dehydrogenase (GAPDH, 1:10,000, ab181602) overnight at 4 °C. Subsequently, membranes were mixed with horseradish peroxidase-conjugated (0.3 µg/mL, ab190492) anti-rabbit antibodies for 1 h at 37 °C. The BeyoECL Plus kit (Beyotime) was used to visualize the protein bands. All the antibodies were obtained from Abcam (Cambridge, MA, USA).

### Cell cycle analysis

Huh-7 and HCCLM3 cells were collected at 48 h after treatment with matrine or transfection, and fixed in 70% ice-cold ethanol overnight at 4 °C. The next day, cells were first treated with 100 μg/mL RNase A and then stained with PI for 30 min in the dark. The distribution of cells at different phases was analyzed using a FACSCalibur flow cytometer (BD Biosciences).

### Transwell assay

Cellular migration and invasion analysis were performed by Transwell assay using 24-well transwell plates (Corning Incorporated, Corning, NY, USA). For invasion detection, Matrigel (Corning Incorporated) needed to be laid at the bottom of the upper chambers in advance. HCC cells were resuspended in serum-free medium, and 100 μL cell suspension was seeded into the upper chambers, while the bottom chambers were added to 600  μL completed DMEM with 10% FBS. 24 h later, cells on the lower side of the chambers were stained with 0.1% crystal violet (Beyotime) for 20 min. After washing with PBS, the stained cells were observed and captured by an inverted microscope. For migration detection, there was no need to add Matrigel in upper chambers, the other steps were the same.

### Quantitative real-time polymerase chain reaction (qRT-PCR)

The RNA from HCC cells or nude mouse tumor tissues was isolated by TRIzol^®^ (Invitrogen). Reverse transcription was performed using the All-in-One™ First-Strand cDNA Synthesis Kit (FulenGen, Guangzhou, China). Then, qRT-PCR was conducted using SYBR Green PCR Master Mix (Applied Biosystems, Foster City, CA, USA) on the 7900HT Fast Real-Time PCR System (Applied Biosystems). The 2^−ΔΔct^ method was utilized to calculate the relative expression, and GAPDH was the internal control for circ_0027345 and HOXD3, while U6 was the internal control for miR-345-5p. The primer sequences were as follows: circ_0027345, F: 5′-TCACTGGTTTGGATGCATTG-3′, R: 5′-AAGGTGGCTCATGGAACTTG-3′. GAPDH, F: 5′-TGATGACATCAAGAAGGTGGTGAAG-3′, R: 5′-TCCTTGGAGGCCATGTGGGCCAT-3′. miR-345-5p, F: 5′-TGAGGGGCAGAGAGCGAGACTTT-3′, R: 5′-CTCAACTGGTGTCGTGGA-3′. U6, F: 5′-ACCCTGAGAAATACCCTCACAT-3′, R: 5′-GACGACTGAGCCCCTGATG-3′. HOXD3, F: 5′-CCATAAATCAGCCGCAAGGAT-3′, R: 5′-GATGGGTCTCAGACTTACCTTTGG-3′.

### Dual-luciferase reporter assay

The sequences of circ_0027345 containing miR-345-5p wild-type (WT) binding sites or mutant (MUT) were cloned into the dual-luciferase reporter vector pmirGLO (Promega, Fitchburg, WI, USA), named as circ_0027345 WT and circ_0027345 MUT. Similarly, the 3′untranslated regions (3′UTRs) of HOXD3 containing miR-345-5p binding sites or mutant were cloned into the pmirGLO vector, named as HOXD3 3′UTR WT and HOXD3 3′UTR MUT. Huh-7 and HCCLM3 cells were harvested at 24 h after co-transfection with miR-345-5p or miR-NC and these vectors, respectively. The luciferase activity was determined using a dual-luciferase reporter assay kit (Promega).

### Tumor xenograft assay

Briefly, suspensions of HCCLM3 tumor cells were inoculated into the 4-week-old male nude mice, and the mice were divided into two groups (n = 5 each group). The mice in treatment group were treated with matrine (50 mg/kg) every day, and mice in control group were treated with same amount of normal saline. The tumor volumes were measured every 5 days. The mice were sacrificed after 30 days of inoculation and tumors were weighed. Furthermore, tumor tissues were preserved at − 80 °C for RNA and protein extraction. The animal experiments were permitted by the Animal Care and Use Committee of Fujian Provincial Hospital.

### Statistical analysis

Data analysis was performed using SPSS v19.0 software, and results were shown as the mean ± standard deviation (SD) from at least three times independently. Student’s *t*-test was utilized to compare the differences between the two groups and one-way analysis of variance (ANOVA) was employed to analyze the differences for multiple comparisons. *P *< 0.05 was considered statistically significant.

## Results

### Matrine inhibited growth, metastasis and activated autophagy of HCC cells in vitro

**Fig. 1:**
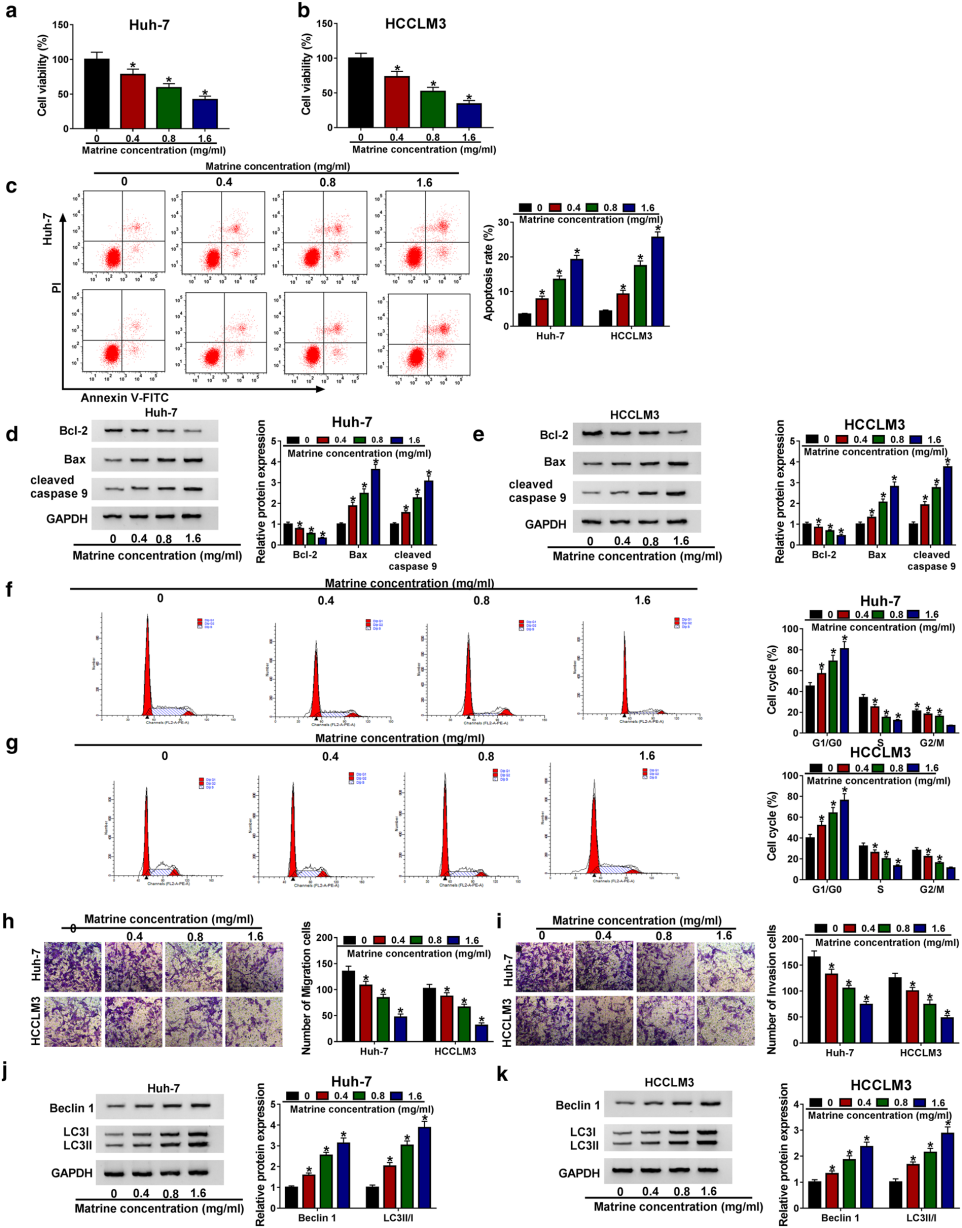
Matrine inhibited growth, metastasis and activated autophagy of HCC cells in vitro. After Huh-7 and HCCLM3 cells were exposed to various concentrations of matrine for 48 h. **a**, **b** Cell viability was determined by CCK-8 assay. **c** Cell apoptosis rate was analyzed by Flow cytometry. **d**, **e** Western blot assay was used to detect the expression of apoptosis-related proteins Bcl-2, Bax and cleaved-caspase 9. **f**, **g** Cell cycle was determined by Flow cytometry. **h**, **i** Cell migration and invasion were estimated by Transwell assay. **j**, **k** Western blot assay was employed to measure the levels of autophagy-related proteins LC3-II, LC3-I and Beclin 1. *P < 0.05

To clarify the role of matrine in HCC, Huh-7 and HCCLM3 cells were treated with various concentrations of matrine for 48 h, following CCK-8 assay was performed. The results showed that matrine repressed cell viability in a dose-dependent manner (Fig. 1a, b). To investigate whether matrine regulated apoptosis of HCC cells, we detected the apoptosis rate by Flow cytometry. As presented in Fig. 1c, the apoptosis ratio of Huh-7 and HCCLM3 cells was significantly increased as the concentration of matrine increasing from 0  mg/mL to 1.6  mg/mL. Bcl-2 and Bax have been widely reported as markers of apoptosis [23]. Western blot data revealed that matrine decreased Bcl-2 expression and increased the expression of Bax in a dose-dependent manner, suggesting that matrine could facilitate apoptosis of Huh-7 and HCCLM3 cells (Fig. 1d, e). Furthermore, Flow cytometry results showed that after the treatment of Huh-7 and HCCLM3 cells with different concentrations of matrine, cell cycle was arrested at the G1/G0 checkpoint, while the cells in S and G2/M checkpoints were decreased (Fig. 1f, g), indicating that matrine could block the cell cycle. Metastasis is the main cause of poor prognosis in cancer patients. To examine whether matrine might repress the migration and invasion of HCC cells, Transwell assay was performed. As presented in Fig. 1h, i, matrine obviously hampered the migration and invasion of Huh-7 and HCCLM3 cells in a dose-dependent manner. Then, the expression of major autophagy regulatory proteins LC3-II, LC3-I and Beclin 1 [24, 25] was measured in matrine-treated Huh-7 and HCCLM3 cells by western blot. The results showed that the expression of Beclin 1 and ratio of LC3-II/I were enhanced in cells treated with matrine in a dose-dependent manner (Fig. 1j, k). These results therefore demonstrated that matrine restrained cell growth, migration, invasion and raised cell apoptosis and autophagy in HCC cells in vitro.

### Matrine suppressed the development of HCC cells by decreasing circ_0027345

**Fig. 2:**
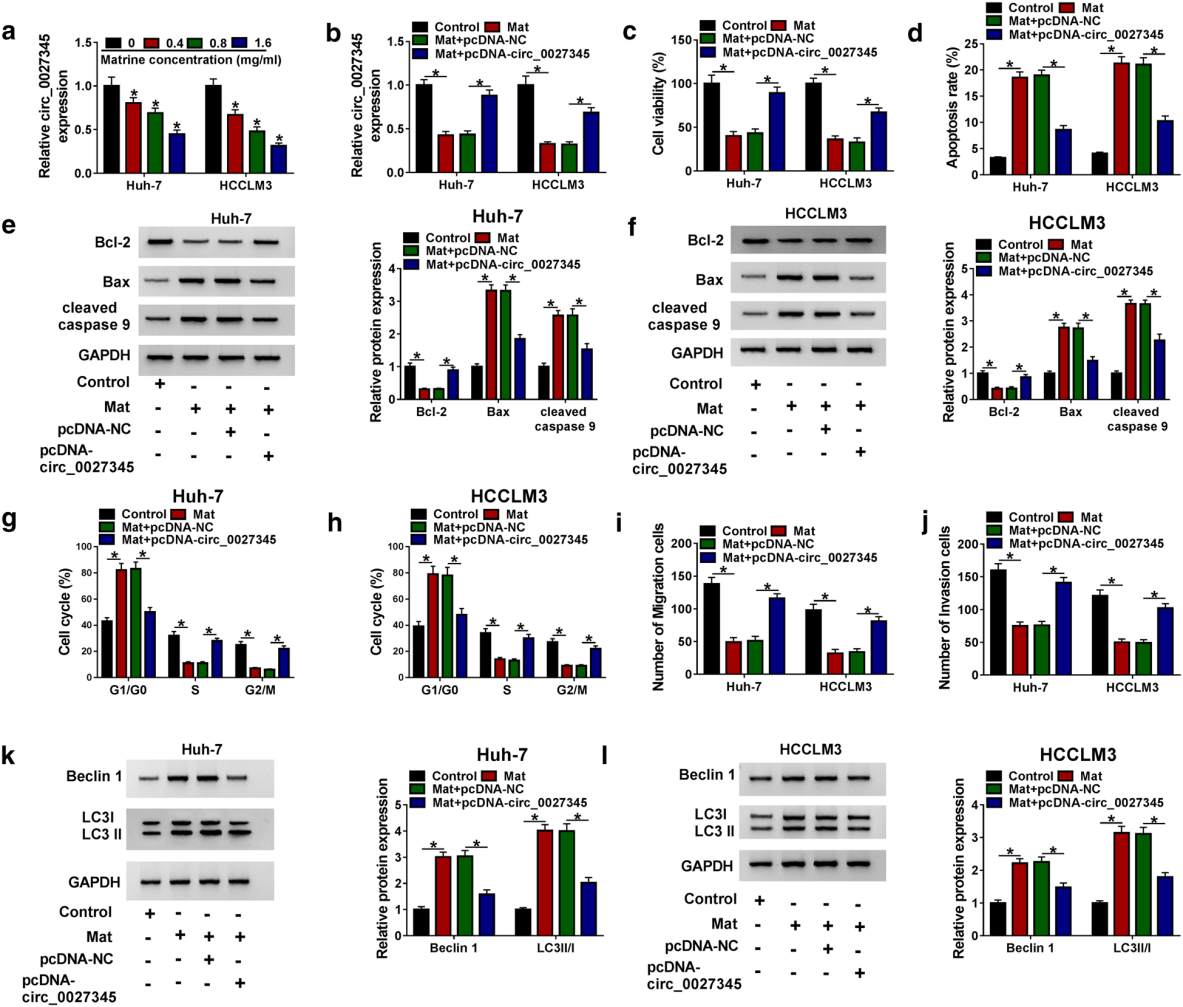
Matrine suppressed the development of HCC cells by decreasing circ_0027345. **a** Huh-7 and HCCLM3 cells were treated with various concentrations of matrine, and circ_0027345 expression was measured by qRT-PCR analysis. **b** Huh-7 and HCCLM3 cells were exposed to different treatments and were divided into the following four groups: control, Matrine (Mat), Mat + pcDNA-NC and Mat + pcDNA-circ_0027345, circ_0027345 expression was then detected by qRT-PCR. **c**, **d** Cell viability and apoptosis were assessed by CCK-8 assay and Flow cytometry, respectively. **e**, **f** The levels of Bcl-2, Bax and cleaved-caspase 9 were examined by western blot. **g**, **j** Cell cycle, migration and invasion were determined by Flow cytometry and Transwell assay, respectively. **k**, **l** LC3-II, LC3-I and Beclin 1 levels were measured by western blot. *P < 0.05

Next, we explored the effect of circ_0027345 on matrine-induced HCC cells, and circ_0027345 expression was first measured. The results of qRT-PCR analysis showed that circ_0027345 expression was evidently decreased in matrine-induced Huh-7 and HCCLM3 cells in a dose-dependent manner (Fig. 2a). As the concentration of 1.6 mg/mL matrine had a high inhibitory effect on circ_0027345 expression, this concentration was used for subsequent experiments. We then overexpressed circ_0027345 in matrine-treated Huh-7 and HCCLM3 cells by transfection of pcDNA-circ_0027345, and the overexpression efficiency was determined by qRT-PCR (Fig. 2b). Subsequently, CCK-8, Flow cytometry and Transwell results showed that overexpression of circ_0027345 could reverse the inhibitory effects of matrine on cell viability (Fig. 2c), cycle (Fig. 2g, h), migration (Fig. 2i) and invasion (Fig. 2j) in Huh-7 and HCCLM3 cells, and also alleviate the promoting impact of matrine on cell apoptosis (Fig. 2d). Besides, the decreased effect of matrine on Bcl-2 and the increased effects on the levels of Bax, cleaved-caspase 9 (Fig. 2e, f), Beclin 1 and LC3-II/LC3-I (Fig. 2k, l) could also be offset by overexpressing circ_0027345. Therefore, we concluded that matrine might inhibit the progression of HCC cells by down-regulating circ_0027345.

### Circ_0027345 was a sponge of miR-345-5p in HCC cells

**Fig. 3:**
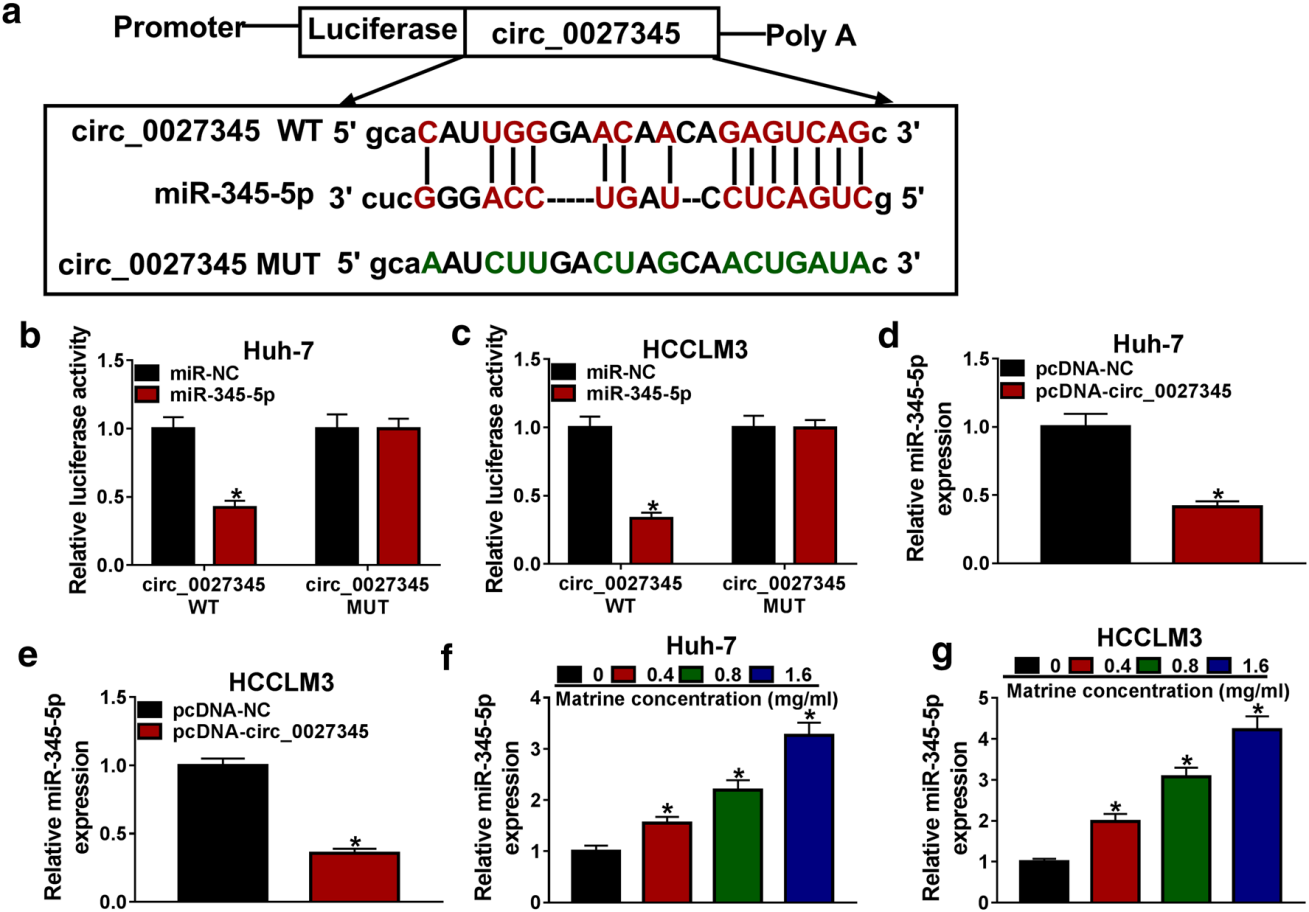
Circ_0027345 was a sponge of miR-345-5p in HCC cells. **a** The binding sites between circ_0027345 and miR-345-5p, and wild-type and mutant of circ_0027345 were displayed. **b**, **c** Dual luciferase reporter assay was performed to clarify the interaction between circ_0027345 and miR-345-5p. **d**, **e** The expression of miR-345-5p in Huh-7 and HCCLM3 cells transfected with pcDNA-NC or pcDNA-circ_0027345 was determined by qRT-PCR. **f**, **g** The expression of miR-345-5p in Huh-7 and HCCLM3 cells treated with various concentrations of matrine was examined by qRT-PCR. *P < 0.05

Increasing evidence has confirmed that circRNAs perform their functions primarily by sponging the target miRNAs [26]. As searched by StarBase v2.0 prediction tool, miR-345-5p contains the binding sites of circ_0027345 (Fig. 3a). Dual luciferase reporter assay was then carried out to confirm the prediction, and the results indicated that the luciferase activity was strikingly reduced in Huh-7 and HCCLM3 cells co-transfected with circ_0027345 WT and miR-345-5p compared to that cells co-transfected with circ_0027345 MUT and miR-345-5p (Fig. 3b, c). Moreover, overexpression of circ_0027345 silenced miR-345-5p expression (Fig. 3d, e). Meanwhile, miR-345-5p expression in matrine-induced Huh-7 and HCCLM3 cells was detected, and qRT-PCR data showed that miR-345-5p expression was elevated in a dose-dependent manner (Fig. 3f, g). These findings supported that circ_0027345 could act as a sponge for miR-345-5p.

### Circ_0027345 overexpression promoted cell progression in matrine-induced HCC cells by reducing miR-345-5p

**Fig. 4:**
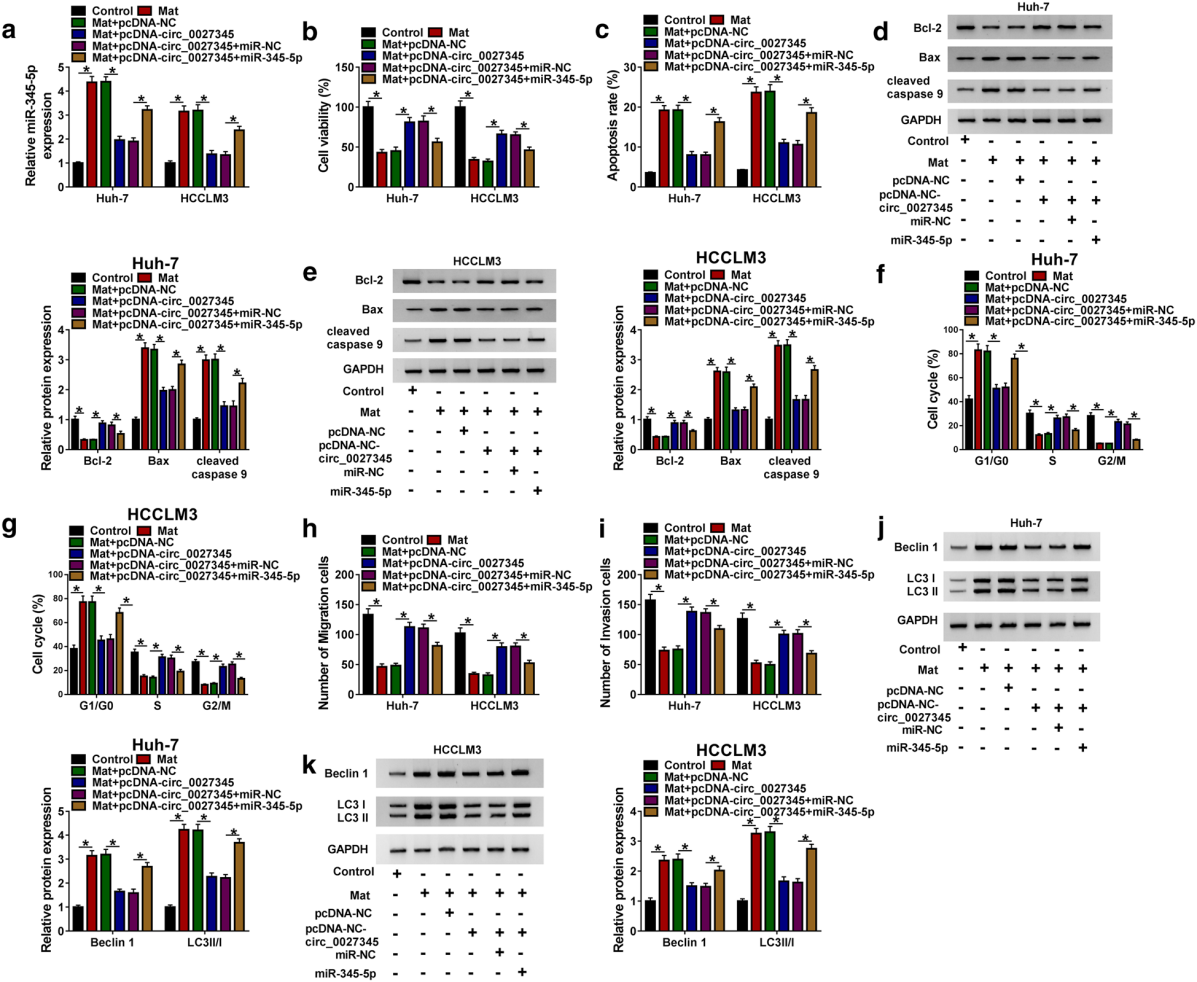
Circ_0027345 overexpression promoted cell progression in matrine-induced HCC cells by reducing miR-345-5p. Huh-7 and HCCLM3 cells were exposed to different treatments and were divided into the following six groups: control, Mat, Mat + pcDNA-NC, Mat + pcDNA-circ_0027345, Mat + pcDNA-circ_0027345 + miR-NC or Mat + pcDNA-circ_0027345 + miR-345-5p. **a** The expression of miR-345-5p was measured by qRT-PCR. **b**, **c** Cell viability and apoptosis were estimated using CCK-8 assay and Flow cytometry, respectively. **d**, **e** The levels of Bcl-2, Bax and cleaved-caspase 9 were detected by western blot. **f**, **g** Cell cycle was evaluated by Flow cytometry. **h**, **i** Cell migration and invasion were analyzed via Transwell assay. **j, k** The levels of LC3-II, LC3-I and Beclin 1 were examined by western blot. *P < 0.05

To investigate whether circ_0027345 could regulate HCC progression by targeting miR-345-5p, pcDNA-circ_0027345 and miR-345-5p mimic (miR-345-5p) were co-transfected into the Huh-7 and HCCLM3 cells exposed to matrine. As shown in Fig. 4a, pcDNA-circ_0027345 and miR-345-5p were transfected into HCC cell lines successfully with high transfection efficiency. CCK-8 and Flow cytometry results revealed that overexpression of circ_0027345 increased cell viability (Fig. 4b) and inhibited cell apoptosis (Fig. 4c) in matrine-treated Huh-7 and HCCLM3 cells, while these effects were reversed by up-regulation of miR-345-5p. As expected, circ_0027345 overexpression enhanced the expression of Bcl-2 and decreased the expression levels of Bax and cleaved-caspase 9, however, miR-345-5p could overturn the trends (Fig. 4d, e). In addition, overexpression of circ_0027345 could accelerate cell cycle progression (Fig. 4f, g), promote cell migration (Fig. 4h) and invasion (Fig. 4i), and inhibit autophagy by down-regulating the levels of Beclin 1 and LC3-II/LC3-I (Fig. 4j, k), all of which could be attenuated by co-transfection of miR-345-5p in matrine-treated Huh-7 and HCCLM3 cells. All the findings implied that circ_0027345 regulated viability, apoptosis, cell cycle, migration, invasion and autophagy by binding to miR-345-5p in matrine-treated HCC cells.

### HOXD3 was the target of miR-345-5p

**Fig. 5:**
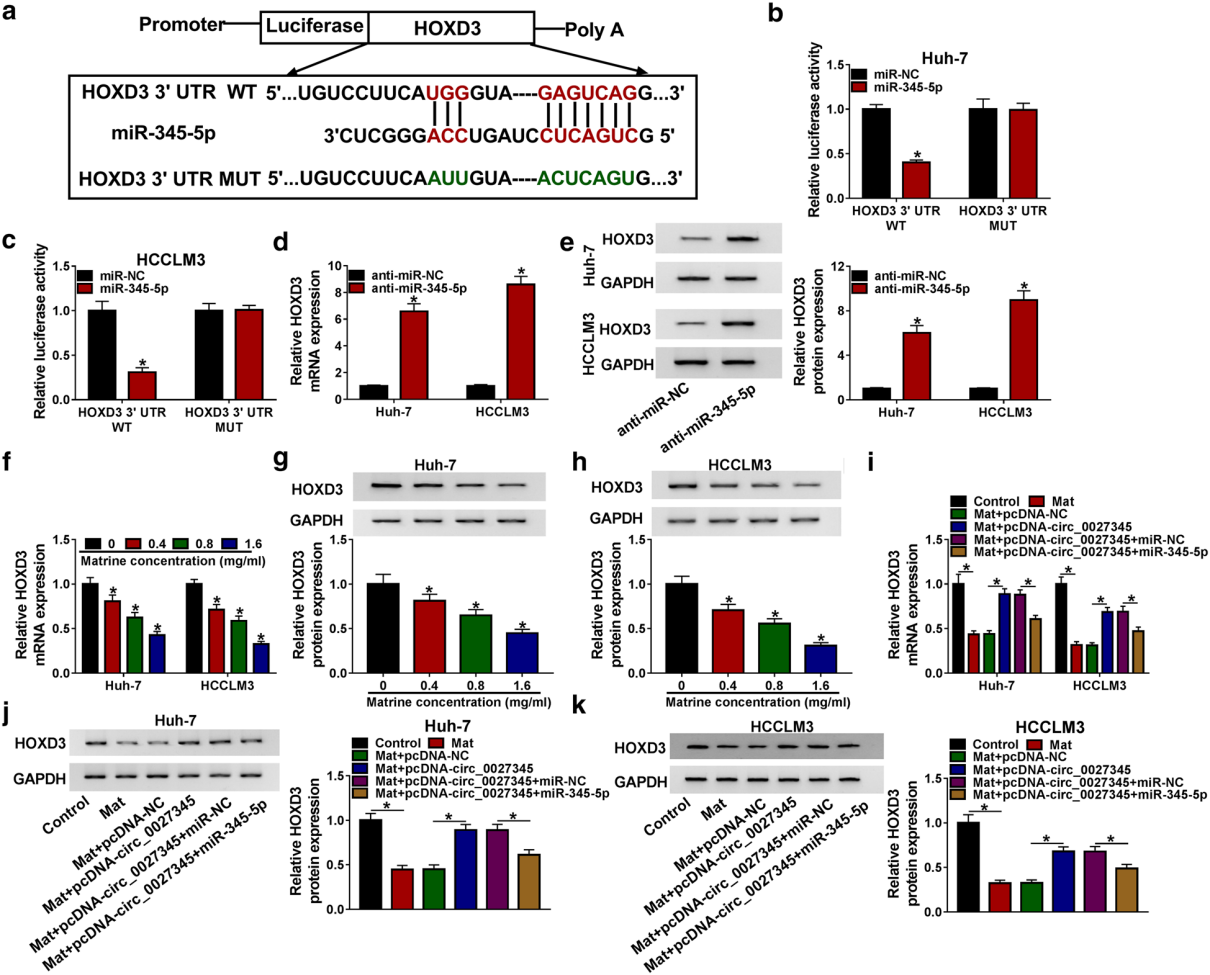
HOXD3 was the target of miR-345-5p. **a** The binding sites between miR-345-5p and HOXD3 were predicted by Targetscan tool. **b**, **c** Dual luciferase reporter assay was performed to confirm the interaction between miR-345-5p and HOXD3. **d**–**e** After Huh-7 and HCCLM3 cells were transfected with anti-miR-NC or anti-miR-345-5p, the mRNA and protein levels of HOXD3 were determined by qRT-PCR and western blot, respectively. **f**–**h** After Huh-7 and HCCLM3 cells were exposed to various concentrations of matrine, the mRNA and protein levels of HOXD3 were measured by qRT-PCR and western blot, respectively. **i**–**k** After Huh-7 and HCCLM3 cells were exposed to different treatments (control, Mat, Mat + pcDNA-NC, Mat + pcDNA-circ_0027345, Mat + pcDNA-circ_0027345 + miR-NC and Mat + pcDNA-circ_0027345 + miR-345-5p), the mRNA and protein levels of HOXD3 were measured by qRT-PCR and western blot, respectively. *P < 0.05

In order to further study the downstream targets of circ_0027345/miR-345-5p axis, Targetscan bioinformatics analysis tool was used to predict the target mRNAs of miR-345-5p. As displayed in Fig. 5a, there were binding sites between miR-345-5p and the 3′UTR of HOXD3. Dual-luciferase reporter assay showed that the luciferase activity was reduced in Huh-7 and HCCLM3 cells co-transfected with miR-345-5p and HOXD3 3′UTR WT compared to miR-345-5p and HOXD3 3′UTR MUT co-transfected group, suggesting the interaction between miR-345-5p and HOXD3 (Fig. 5b, c). Additionally, the mRNA and protein levels of HOXD3 were enhanced by anti-miR-345-5p in Huh-7 and HCCLM3 cells (Fig. 5d, e). As expected, HOXD3 expression was dwindled in matrine-stimulated Huh-7 and HCCLM3 cells in a dose-dependent manner at mRNA and protein levels (Fig. 5f–h). More importantly, in matrine-treated Huh-7 and HCCLM3 cells, circ_0027345 overexpression could augment the mRNA (Fig. 5i) and protein levels of HOXD3 (Fig. 5j, k), while this effect could be weakened by overexpressing miR-345-5p. The above results supported that circ_0027345 acted as a ceRNA of miR-345-5p to regulate HOXD3 expression.

### HOXD3 knockdown reversed the promoting effects of anti-miR-345-5p on cell progression in matrine-induced HCC cells

**Fig. 6:**
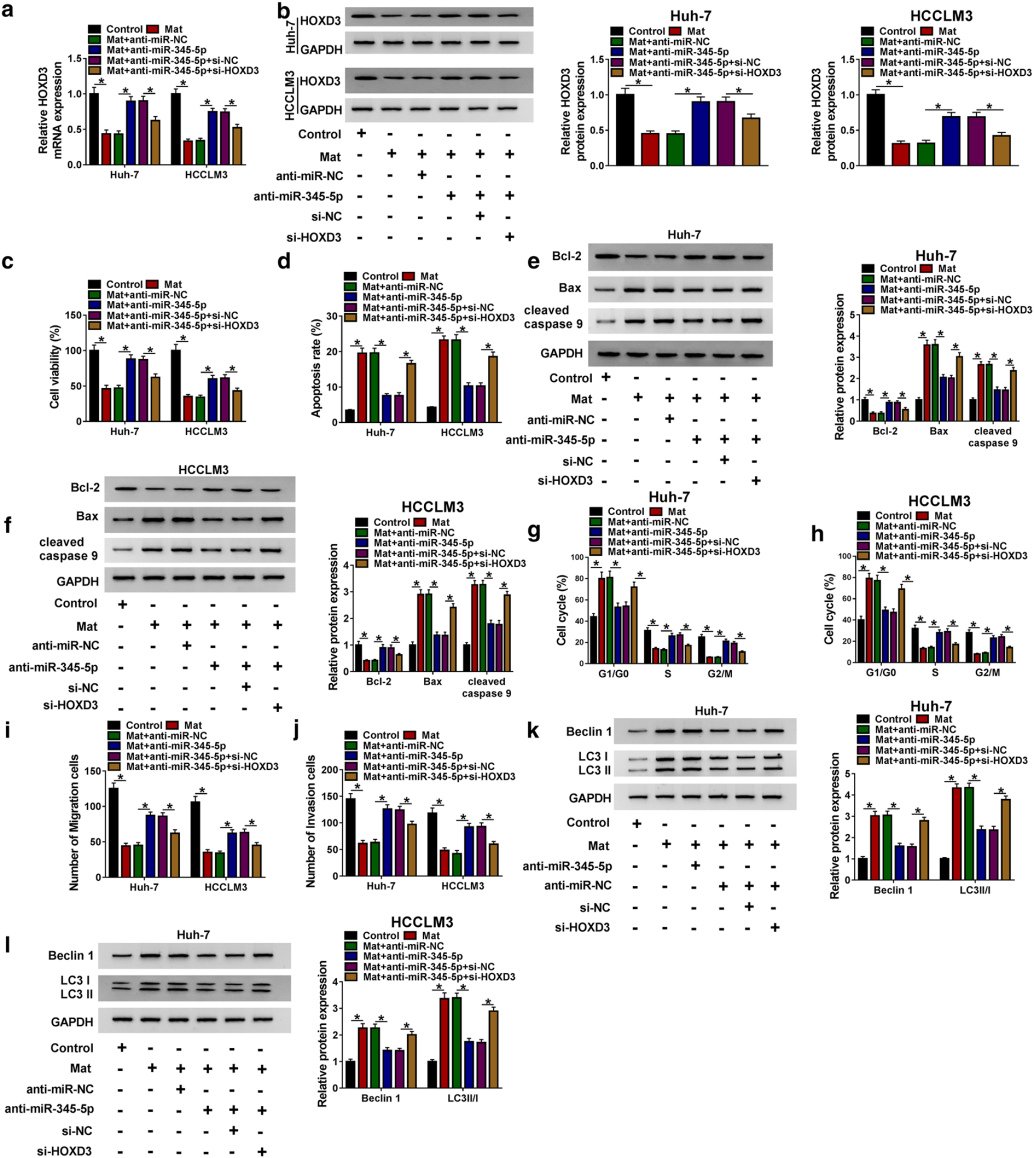
HOXD3 knockdown reversed the promoting effects of anti-miR-345-5p on cell progression in matrine-induced HCC cells. After Huh-7 and HCCLM3 cells were exposed to different treatments (control, Mat, Mat + anti-miR-NC, Mat + anti-miR-345-5p, Mat + anti-miR-345-5p + si-NC and Mat + anti-miR-345-5p + si-HOXD3). **a**, **b** The mRNA and protein levels of HOXD3 were measured by qRT-PCR and western blot, respectively. **c**, **d** Cell viability and apoptosis were examined through CCK-8 assay and Flow cytometry, respectively. **e**, **f** Western blot assay was used to measure the levels of Bcl-2, Bax and cleaved-caspase 9. **g**–**j** Cell cycle, migration and invasion were assessed by Flow cytometry and Transwell assay, respectively. **k**–**l** Western blot assay was performed to evaluate the levels of LC3-II, LC3-I and Beclin 1. *P < 0.05

After confirming the targeted relationship between miR-345-5p and HOXD3, we further explored whether HOXD3 was involved in the regulation of miR-345-5p on HCC cell progression. As shown in Fig. 6a, b, co-transfection of si-HOXD3 counteracted the promotion effect of anti-miR-345-5p on mRNA and protein levels of HOXD3 in matrine-treated Huh-7 and HCCLM3 cells. As expected, repression of miR-345-5p potentiated cell viability (Fig. 6c) and impaired cell apoptosis (Fig. 6d), and these effects were restored by silencing HOXD3. Similarly, the effect of miR-345-5p knockdown on expression of apoptotic markers Bcl-2, Bax and cleaved-caspase 9 (Fig. 6e, f) could also be rescued by interfering with HOXD3. More than that, the facilitated effects of anti-miR-345-5p on cycle (Fig. 6g, h), migration (Fig. 6i) and invasion (Fig. 6j) were also abrogated by HOXD3 deficiency in matrine-stimulated Huh-7 and HCCLM3 cells. Meanwhile, the inhibitory effects of anti-miR-345-5p on levels of Beclin 1 and LC3-II/LC3-I were reversed by repression of HOXD3 (Fig. 6k, l). The results indicated that miR-345-5p could regulate cell development in matrine-stimulated HCC cells through targeting HOXD3.

### Matrine inhibited the tumor growth of HCC in vivo

**Fig. 7:**
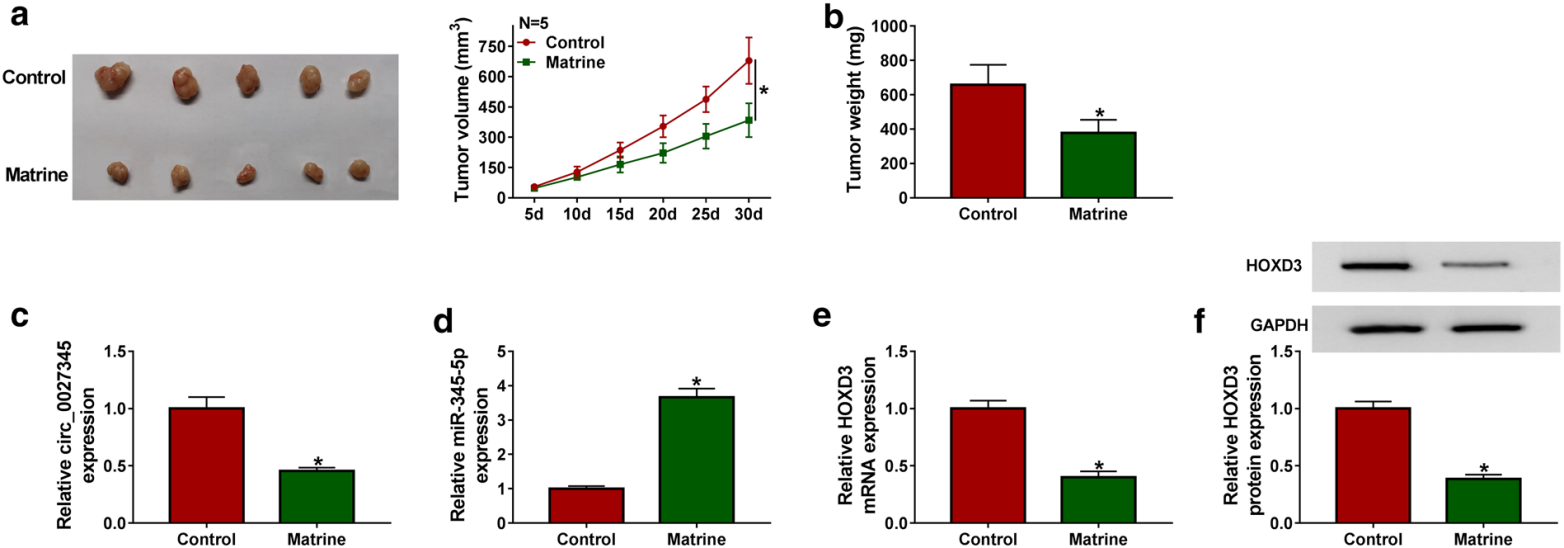
Matrine inhibited the tumor growth of HCC in vivo. HCCLM3 cells were inoculated into nude mice and divided into two groups (n = 5 each group), one treated with matrine and the other treated with saline to act as control. **a**, **b** Volume and weight of the tumors were measured. **c**–**e** Abundances of circ_0027345, miR-345-5p and HOXD3 in that tumor tissues were examined by qRT-PCR. **f** The protein expression of HOXD3 was determined by western blot. *P < 0.05

According to the above results, matrine could hamper the development of HCC cells in vitro, we further evaluated whether matrine had the same inhibitory effect in vivo. To verify this guess, a mouse xenograft model was constructed. The results were shown in Fig. 7a, b, the tumors of mice treated with matrine had smaller volume and weight. Consistent with the above results in vitro, circ_0027345 expression (Fig. 7c) and the mRNA and protein levels of HOXD3 (Fig. 7e, f) were substantially decreased in tumor tissues of matrine-treated group, and miR-345-5p expression was markedly increased (Fig. 7d). The data in vivo proved that matrine hindered the tumor growth of HCC by up-regulating miR-345-5p and down-regulating circ_0027345 and HOXD3.

## Discussion

HCC, which has high morbidity and mortality rate and lacks effective therapeutic drugs [27]. Recently, the anti-tumor effect of traditional Chinese medicine matrine has attracted wide attention. Research has shown that matrine impeded cell metastasis, and elevated cell apoptosis and autophagy in HCC cells [28, 29]. In line with these results, we found that matrine could impair cell viability, migration and invasion. Besides, matrine accelerated cell apoptosis by reducing Bcl-2 and increasing Bax, at the same time, matrine promoted cell autophagy by enhancing LC3-II/LC3-I and Beclin 1. Bcl-2 and Bax are identified as anti-apoptotic and pro-apoptotic proteins respectively [30]. Beclin 1 is a key protein in the formation of autophagosome [31]. Autophagy is a catabolic mechanism, and its changes in activity are a double-edged sword to the growth of tumor cells, which can maintain body development, aging and degeneration [32]. Moreover, LC3-I is transformed to LC3-II during autophagy, so, the raise of LC3-II/LC3-I ratio indicates the improvement of autophagy level [33]. These results showed that matrine had anti-tumor effect, which could not only reduce cell activity, inhibit cell migration and invasion, but also induce apoptosis and autophagy.

As a novel RNA molecule, circRNA can regulate the physiological and pathological processes of various cancers, including HCC [34]. Others like Su et al. revealed that circRNA Cdr1 could facilitate cell proliferation, migration and tumor growth by acting as a ceRNA of miR-1270 [35]. In this study, we demonstrated that circ_0027345 was down-modulated in matrine-treated HCC cells, and overexpression of circ_0027345 could reverse the effects of matrine on HCC cells, suggesting that matrine exerted its anti-tumor effects by silencing circ_0027345 in HCC cells.

CircRNAs have been shown to interact with miRNAs to regulate tumor progression by acting as a sponge for miRNA in HCC [36]. MiR-345-5p was identified to be a target of circ_0027345 in this work. Coincidentally, miR-345-5p was supported to be a gene with cell migration and invasion-associated in several tumors [14]. In addition, miR-345 could restrain tumor metastasis in HCC [37]. Our data showed that matrine could elevate miR-345-5p expression, implying that matrine might suppress the progression of HCC by increasing miR-345-5p. To verify our hypothesis, the recovery experiments were carried out. The results indicated that up-regulation of miR-345-5p counteracted the promotion effect of circ_0027345 on cell progression in matrine-induced HCC cells, confirming that matrine could raise miR-345-5p expression through circ_0027345 to play an anti-cancer role in HCC cells.

HOXD3 is an important transcription factor for maintaining cell structure and regulating cell motility [20]. Zhu et al. revealed that circRNA PVT1 could increase HOXD3 expression by serving as a ceRNA of miR-203, thus promoting the growth, migration and tumorigenesis of HCC cells [38]. Interestingly, miR-345-5p directly targeted HOXD3, and miR-345-5p regulated the development of HCC cells by targeting HOXD3. Then, we analyzed the interactions among circ_0027345, miR-345-5p, and HOXD3. The findings revealed that circ_0027345 could act as miR-345-5p sponge to augment HOXD3 expression. From the above data, we concluded that the anti-cancer effects of matrine might be achieved by increasing miR-345-5p and decreasing circ_0027345 and HOXD3 in vitro. Consistent with the results in vitro, matrine could inhibit tumor growth in vivo by inducing miR-345-5p and reducing circ_0027345 and HOXD3.

## Conclusion

Collectively, our results suggested that matrine suppressed cell growth, metastasis and induced apoptosis and autophagy by up-regulation of miR-345-5p and down-regulation of circ_0027345 and HOXD3. This study laid a foundation for further evaluation of matrine as a clinical therapy for HCC, and provided a new molecular regulation mechanism such as circ_0027345/miR-345-5p/HOXD3.

## Data Availability

The analyzed data sets generated during the present study are available from the corresponding author on reasonable request.

## Abbreviations

HCC: Hepatocellular carcinoma
CCK-8: Cell counting kit-8
HOXD3: Homeobox-containingD3
qRT-PCR: Quantitative real-time polymerase chain reaction
SDS-PAGE: Sodium dodecyl sulfate–polyacrylamide gel electrophoresis
WT: Wild-type
MUT: Mutant
SD: Standard deviation
ANOVA: Analysis of variance

## References

[1] Berkan-Kawinska A (2019). Hepatocellular carcinoma in non-alcohol fatty liver disease—changing trends and specific challenges. Curr Med Res Opin.

[2] Jemal A (2011). Global cancer statistics. CA Cancer J Clin.

[3] Bruix J (2002). Prognostic prediction and treatment strategy in hepatocellular carcinoma. Hepatology.

[4] Huang WC (2014). Matrine attenuates allergic airway inflammation and eosinophil infiltration by suppressing eotaxin and Th2 cytokine production in asthmatic mice. J Ethnopharmacol.

[5] Zhang YB (2018). Matrine-type alkaloids from the roots of sophora flavescens and their antiviral activities against the hepatitis B virus. J Nat Prod.

[6] Wei YP (2018). Matrine exerts inhibitory effects in melanoma through the regulation of miR-19b-3p/PTEN. Int J Oncol.

[7] Zhou W (2018). Matrine induces senescence of human glioblastoma cells through suppression of the IGF1/PI3K/AKT/p27 signaling pathway. Cancer Med.

[8] Zhao L (2018). Matrine inhibits TPC-1 human thyroid cancer cells via the miR-21/PTEN/Akt pathway. Oncol Lett.

[9] Wang Y (2018). Matrine inhibits the invasive and migratory properties of human hepatocellular carcinoma by regulating epithelialmesenchymal transition. Mol Med Rep.

[10] Li Y (2015). Circular RNA is enriched and stable in exosomes: a promising biomarker for cancer diagnosis. Cell Res.

[11] Guarnerio J (2016). Oncogenic role of fusion-circRNAs derived from cancer-associated chromosomal translocations. Cell.

[12] You X (2015). Neural circular RNAs are derived from synaptic genes and regulated by development and plasticity. Nat Neurosci.

[13] Sun S (2019). Circular RNA circ-ADD3 inhibits hepatocellular carcinoma metastasis through facilitating EZH2 degradation via CDK1-mediated ubiquitination. Am J Cancer Res.

[14] Mou T (2019). MiR-345-5p functions as a tumor suppressor in pancreatic cancer by directly targeting CCL8. Biomed Pharmacother.

[15] Yu J (2019). E2F1-induced upregulation of long non-coding RNA LMCD1-AS1 facilitates cholangiocarcinoma cell progression by regulating miR-345-5p/COL6A3 pathway. Biochem Biophys Res Commun.

[16] Zhang H (2017). MicroRNA-345 inhibits hepatocellular carcinoma metastasis by inhibiting YAP1. Oncol Rep.

[17] Jia N (2019). CeRNA expression profiling identifies KIT-related circRNA-miRNA-mRNA networks in gastrointestinal stromal tumour. Front Genet.

[18] Hutlet B (2016). Systematic expression analysis of Hox genes at adulthood reveals novel patterns in the central nervous system. Brain Struct Funct.

[19] Zhang B (2018). Knockdown of homeobox B5 (HOXB5) inhibits cell proliferation, migration, and invasion in non-small cell lung cancer cells through inactivation of the Wnt/beta-catenin pathway. Oncol Res.

[20] Hamada J (2001). Overexpression of homeobox gene HOXD3 induces coordinate expression of metastasis-related genes in human lung cancer cells. Int J Cancer.

[21] Zhang Y (2018). HOXD3 plays a critical role in breast cancer stemness and drug resistance. Cell Physiol Biochem.

[22] Wang L (2018). HOXD3 targeted by miR-203a suppresses cell metastasis and angiogenesis through VEGFR in human hepatocellular carcinoma cells. Sci Rep.

[23] Chipuk JE (2004). Direct activation of Bax by p53 mediates mitochondrial membrane permeabilization and apoptosis. Science.

[24] Kuo SH (2012). Macroautophagy abnormality in essential tremor. PLoS ONE.

[25] Naguib M (2018). Serum level of the autophagy biomarker Beclin-1 in patients with diabetic kidney disease. Diabetes Res Clin Pract.

[26] Song W (2019). Circular RNA-associated competing endogenous RNA network and prognostic nomogram for patients with colorectal cancer. Front Oncol.

[27] Couri T (2019). Goals and targets for personalized therapy for HCC. Hepatol Int.

[28] Zhang J (2019). Matrine suppresses lung metastasis of human hepatocellular carcinoma by directly targeting matrix metalloproteinase-9. Biochem Biophys Res Commun.

[29] Xie SB (2015). Matrine-induced autophagy regulated by p53 through AMP-activated protein kinase in human hepatoma cells. Int J Oncol.

[30] Walensky LD (2019). Targeting BAX to drug death directly. Nat Chem Biol.

[31] Pattingre S (2005). Bcl-2 antiapoptotic proteins inhibit Beclin 1-dependent autophagy. Cell.

[32] Wang Z (2014). Autophagy in kidney health and disease. Antioxid Redox Signal.

[33] Tanida I (2004). LC3 conjugation system in mammalian autophagy. Int J Biochem Cell Biol.

[34] Zhang HD (2018). CircRNA: a novel type of biomarker for cancer. Breast Cancer.

[35] Su Y (2019). CircRNA Cdr1as functions as a competitive endogenous RNA to promote hepatocellular carcinoma progression. Aging (Albany NY).

[36] Liu Z (2019). CircRNA-5692 inhibits the progression of hepatocellular carcinoma by sponging miR-328-5p to enhance DAB2IP expression. Cell Death Dis.

[37] Yu M (2017). miR-345 inhibits tumor metastasis and EMT by targeting IRF1-mediated mTOR/STAT3/AKT pathway in hepatocellular carcinoma. Int J Oncol.

[38] Zhu Y (2019). The circular RNA PVT1/miR-203/HOXD3 pathway promotes the progression of human hepatocellular carcinoma. Biol Open.

